# Supplementary material for: Adherence to a Supplemented Mediterranean Diet Drives Changes in the Gut Microbiota of HIV-1-Infected Individuals
Source: Nutrients. 2021 Mar 30;13(4):1141. doi: 10.3390/nu13041141 (PMC8067262; doi:10.3390/nu13041141)
Supplement: Supplementary file 1 [file nutrients-13-01141-s001.pdf]

Supplementary Tables

Supplementary Table 1. Characteristics of study cohort at baseline (82 participants). *Median (IQR). **n (%). Acronyms: MSM, man who have sex with men; ART, anti-retroviral therapy; ITINAN, non-nucleoside reverse transcriptase inhibitors; IIN: integrase inhibitors; IP: protease inhibitors; BMI, body mass index. The unique transgender female present in the cohort was considered as MSM. No statistical differences were found for any of the presented parameters between randomization groups.

Group	Classification	All dataset	Control group	SMD group
	n subjects	82	42	40
**Sex** **	Male	69 (84 %)	35 (83 %)	34 (85 %)
	Female	12 (15 %)	6 (14 %)	6 (15 %)
	Transgender female	1 (1 %)	1 (2 %)	0
**Risk group** **	MSM	60 (73 %)	33 (79 %)	27 (68 %)
	no-MSM	20 (25 %)	9 (21 %)	11 (27 %)
**Origin** **	Unknown	2 (2 %)	0	2 (5 %)
	Spain	44 (54 %)	23 (55 %)	21 (53 %)
	South and central America	11 (13 %)	5 (12 %)	6 (15 %)
	Subsaharian Africa	1 (1 %)	1 (2 %)	0
	Europe	3 (4 %)	1 (2 %)	2 (5 %)
	Unknown	23 (28 %)	12 (29 %)	11 (27 %)
**Age** *		47 (40, 53)	47.5 (39, 52)	46 (42, 53)
**Years on cART** *		12 (8, 17)	13 (8, 16)	12 (8, 19)
**ART type** **	ITINAN	23 (28 %)	7 (17 %)	16 (40%)
	IIN	53 (65 %)	30 (71 %)	23 (58 %)
	IP	5 (6 %)	4 (10 %)	1 (2 %)
	other	1 (1%)	1 (2 %)	0
**BMI** *		26 (23, 28)	25 (23, 27)	26 (24, 29)
**BMI classification** **	Overweight	7 (9 %)	3 (7 %)	4 (10 %)
	High	42 (51 %)	21 (50 %)	21 (52 %)
	Normal	28 (34 %)	16 (38 %)	12 (30 %)
	Low	1 (1 %)	0	1 (3 %)
	Unknown	4 (5 %)	2 (5 %)	2 (5 %)
**CD4-nadir (cell/mm3)** *		378 (312, 468)	355 (296, 418)	392 (336, 499)
**CD4 (cell/mm3)** *		820 (663, 1028)	831 (680, 1035)	768 (634, 999)

Supplementary Table 2. Metabolic, inflammation, bacterial translocation, immunological and nutrition markers in Supplemented Mediterranean Diet group (SMD) and control group. All individuals with complete data were selected (n = 82). Data shown are average by group at baseline and week 12. Mann-Whitney-Wilcoxon test with Bonferroni was used to examine parameters between Control and SMD groups. *P value for the difference between baseline and week 12 values (paired). **P value for the difference between delta and ratios values of both groups (unpaired). P<0.05 was considered significant and highlighted in bold type. Acronyms: Hdl, high-density lipoprotein; Ldl, low-density lipoprotein; uRCP, ultrasensitive c-reactive protein; IL-6, interleukin-6; DD, D-dimer; sCD14, soluble CD14; LBP, lipopolysaccharide binding-protein; Treg, regulatory T-cell; BMI, body mass index; IFNg, interferon gamma.

		**Control group**			**SMD group**			**Δ Controls vs SMD**
**Parameters**	**Markers**	**Baseline**	**week12**	**P ***	**Baseline**	**week12**	**P ***	**P ****
**Metabolic**								
	**Glucose (mg/dL)**	85.5 (77, 92.75)	85.5 (75.5, 93)	0.65	89 (79, 94.25)	95 (83, 103.75)	0.07	0.33
	**Creatinine (mg/dL)**	0.9 (0.79, 1.06)	0.86 (0.81, 1)	0.86	0.93 (0.80, 1.05)	0.91 (0.79, 0.97)	0.14	0.4
	**Cholesterol (mg/dL)**	179.5 (156.5, 208)	195.5 (165.8, 211.5)	**0.05**	181.5 (154, 205.02)	173 (156.8, 197.2)	0.739	**0.025**
	**Hdl (mg/dL)**	40 (34, 46.75)	40.5 (35, 48)	0.26	39 (34, 46.5)	41 (35, 48.25)	0.3	0.99
	**Ldl (mg/dL)**	116 (87.25, 139.75)	129 (101, 144.8)	0.49	115 (91.5, 133)	117 (88, 131.5)	0.43	0.24
	**A1-lipoprotein (mg/dL)**	131 (124.5, 141.5)	131.5 (121.8, 145.2)	0.25	131 (119.5, 141)	128 (118.5, 139.5)	0.63	0.37
	**B-lipoprotein (mg/dL)**	98 (84, 113.5)	107.5 (92.75, 121.25)	**0.02**	94 (82.5, 116)	98 (82,110)	0.72	**0.05**
	**Triglycerides (mg/dL)**	114.5 (73, 163.5)	109 (84.25, 136.5)	0.81	118 (71, 145.2)	94 (74.5, 153.2)	0.91	0.77
	**Alkaline phosphatase (U/L)**	75.5 (70, 94.75)	73.5 (64.5, 87.5)	0.52	77.5 (69, 95.5)	73 (65.75, 99)	0.97	0.55
	**Asat (U/L)**	24 (21, 27)	23 (21, 28.75)	0.14	27.5 (22.75,30.25)	25.5 (22, 29.25)	0.33	0.06
	**Alat (U/L)**	22.5 (17, 29.25)	21.5 (18, 30)	0.22	26.5 (19.25, 32,25)	28.93 (27.5, 33.25)	0.82	0.64
	**Bilirubin-total (mg/dL)**	0.7 (0.4, 0.87)	0.6 (0.4, 0.7)	0.16	0.5 (0.4, 0.63)	0.5 (0.4, 0.7)	0.25	1
	**Platelets (10^9/L)**	214 (186.5, 251.5)	228 (202, 256)	0.13	248 (204, 270.3)	243 (208.5, 276)	0.68	0.53
	**Leucocytes (10^9/L)**	6.5 (5.45, 7.84)	6.37 (5.5, 7.16)	0.14	6.63 (5.86, 7.94)	6 (5.46, 7.19)	0.12	0.78

	**Hematocrit (L/L)**	0.44 (0.42, 0.46)	0.45 (0.43, 0.47)	0.8	0.45 (0.42, 0.46)	0.44 (0.44, 0.46)	0.97	0.63
**HIV infection**								
	**Viral load (cp/mL)**	undetectable	undetec	-	undetec	undetect	-	–
	**CD4+ T-cells (cell/mm3)**	825.5 (680, 1032.8)	801 (682, 965)	0.33	761 (605.5, 994.2)	688.5 (591, 986.2)	0.76	0.49
**Inflammation**								
	**uRCP (mg/dL)**	0.17 (0.07, 0.38)	0.15 (0.06, 0.34)	0.64	0.12 (0.08, 0.19)	0.15 (0.09, 0.43)	0.08	0.04
	**IL-6 (pg/dL)**	2 (2, 3)	2 (2, 3)	0.84	2 (2, 4)	2 (2, 4)	0.9	0.81
	**DD (ng/dL)**	200 (200, 300)	200 (200, 300)	0.84	200 (200, 400)	200 (200, 300)	0.9	0.81
**Bacterial translocation**								
	**sCD14 (ng/dL)**	1710 (1534, 1933)	1709 (1557, 2489)	0.83	1706 (1515.2, 1834,3)	1742.8 (1538, 1848.9)	0.22	0.25
	**LBP (ng/dL)**	12016 (10266, 15876)	12455 (9713, 14152)	0.83	10142 (8768, 12199)	11075 (9238, 13603)	**0.05**	0.26
	**Endocab (MMU/mL)**	41.15 (25.35, 60.60)	51.2 (26.25, 78.15)	0.05	51.5 (35.46, 75.03)	40.52 (30.62, 120.05)	0.96	0.11
**Immune activation**								
	**CD4+ (%)**	51.17 (42.22, 59.26)	51.89 (44.34, 57.33)	0.63	44.93 (40.56, 50.94)	48.36 (40.88, 52.67)	0.07	0.32
	**CD8+ (%)**	39.70 (31.75, 48.09)	40.61 (34.02, 48.31)	0.63	46.48 (41.15, 52.30)	44.54 (38.82, 53.19)	0.11	0.42
	**CD4+HLADR+CD38+ (%)**	1.130 (0.80, 1.82)	1.04 (0.79, 2.00)	0.57	1.42 (0.76, 2.14)	1.22 (0.85, 1.85)	0.18	0.15
	**CD8+HLADRCD+38+ (%)**	3.46 (1.930, 5.74)	3.40 (2.28, 5.51)	0.29	2.99 (2.17, 5.49)	2.85 (1.97, 4.78)	0.29	0.11
**Treg cells**								
	**CD4+Foxp3+CD25+ (%)**	4.33 (3.5, 5.56)	3.91 (3.26, 5.17)	0.26	4.36 (3.40, 4.81)	4.41 (3.48, 5.4)	0.38	0.48
	**CD4+Foxp3+CD25+bright (%)**	2 (1.53, 2.7)	1.8 (1.53, 2.41)	0.23	1.98 (1.58, 2.25)	1.99 (1.4, 2.76)	0.65	0.29
	**CD4+Foxp3+CD25- (%)**	3.67 (3.24, 4.35)	3.5 (2.98, 4.36)	0.56	3.74 (3.09, 4)	3.85 (3.05, 5.03)	0.96	0.91
	**CD4+Foxp3+CD25+CD127- (%)**	3.69 (3.1, 5.1)	3.4 (2.77, 4.76)	0.16	3.73 (2.99,3.83)	3.92 (3.03, 4.49)	0.36	0.28
	**CD4+ Foxp3+CD25+CD127+ (%)**	0.44 (0.35, 0.65)	0.52 (0.33, 0.68)	0.46	0.51 (0.41, 0.72)	0.51 (0.37, 0.69)	0.54	0.28
	**CD4+Foxp3+CD25+brightCD127- (%)**	1.81 (1.37, 2.37)	1.64 (1.24, 2.21)	0.16	1.81 (1.37, 2.1)	1.82 (1.25, 2.5)	0.49	0.27
	**CD4+ Foxp3+CD25+brightCD127+ (%)**	0.14 (0.1, 0.25)	0.18 (0.1, 0.22)	0.39	0.18 (0.11, 0.23)	0.16 (0.11, 0.22)	0.65	0.51
	**CD4+Foxp3+CD25-CD127+ (%)**	32.27 (28.96, 37.23)	36.73 (29.94, 40.7)	0.16	38.39 (33.9, 45.94)	37.1 (29.23, 47.43)	0.6	0.15
**T-helper 17 (Th17) cells**								
	**CD4+IL17A+ (%)**	0.64 (0.44, 0.83)	0.6 (0.5, 0.85)	0.81	0.73 (0.48, 0.99)	0.72 (0.43, 1.02)	0.94	0.83

	**CD4+IFNg+ (%)**	20. 24 (15.86, 26.49)	20. 36 (16.32, 28.6)	0.57	18 (13.4, 24.74)	18.07 (12.99, 22.25)	0.09	0.07
	**CD4+IL17A+IFNg+ (%)**	0.12 (0.07, 0.17)	0.12 (0.07, 0.17)	0.52	0.11 (0.07, 0.24)	0.1 (0.07, 0.24)	0.46	0.34
	**CD8+IL17A+ (%)**	0.01 (0.01, 0.03)	0.02 (0.01, 0.04)	0.93	0.02 (0.01, 0.03)	0.02 (0.01, 0.03)	0.97	0.8
	**CD8+IFNg+ (%)**	63.1 (44.2, 76.17)	61.56 (48.94, 75.41)	0.65	61.51 (49.51, 69.35)	50.49 (40.93, 68.1)	**< 0.005**	**0.01**
	**CD8+IL17A+IFNg+ (%)**	0.02 (0.01, 0.04)	0.02 (0.01, 0.04)	0.86	0.02 (0.01, 0.05)	0.02 (0.01, 0.04)	**0.05**	**0.04**
**Nutrition**								
	**BMI**	25 (23, 27)	25 (24, 27)	1	26 (15, 28.75)	26 (24, 28)	0.86	0.39
	**Weight (Kg)**	74.2 (68.28, 80.1)	74.25 (69.5, 80.23)	0.97	74.75 (67.88, 83.12)	74,35 (67.78, 82.6)	0.93	1
	**Adherence (MEDAS)**	8 (5,9)	8 (6,9)	1	6 (5, 8)	12 (10, 13)	**< 0.005**	**< 0.005**
	**Oleic (g/dL)**	20.22 (15.28, 25.48)	20.45 (14.95, 25.58)	0.5	22.01 (19.2, 25.48)	20.66 (18.07, 24.1)	0.41	0.27
	**Linoleic (g/dL)**	22 (20.11, 25.54)	22.2 (19.44, 26.84)	0.33	24.15 (21.67, 28.74)	25.27 (21.23, 30.85)	0.12	0.41
	**Alfa-linolenic (g/dL)**	0.32 (0.2, 0.45)	0.31 (0.2, 0.45)	0.1	0.3 (0.27, 0.5)	0.54 (0.35, 1.15)	**< 0.005**	**< 0.005**

Supplementary Table 3. Key food items and dietary nutrients intake in MSM individuals grouped by their MEDAS at week 12. Baseline values and changes in dietary nutrients intake and key food items using the PREDIMED 14-point Mediterranean diet questionnaire. MSM individuals (n = 60) were stratified by the MEDAS after 12 weeks of follow-up. 2 participants were not considered for lack of MEDAS data at week 12. * Kruskal-Wallis test with Bonferroni was used to examine parameters Adherence groups: P-value < 0.05. a: P<0.05 in Low vs Medium; b: P<0.05 in Low vs High. P<0.05 was considered significant and highlighted in bold type.

			**Adherence to the MD**			
**Diet Parameters**	**Markers**	**Time-point**	**Low (n = 8)**	**Medium (n = 20)**	**High (n = 30)**	**P**
**Dietary nutrients intake**						
	**EVOO, g/d**	Baseline	20.83±24.58	32.14±17.93	34.04±13.19	
		Week12	33.33±25.82	35.71±18.66 a	53.33±7.61*	**0.002**
	**Refined OO, g/d**	Baseline	8.33±12.91	11.67±19.58	3.85±9.2	
		Week12	4.17±10.21	10.48±19.49	0±0*	0.437
	**Total nuts, g/d**	Baseline	7.05±8.1	21.25±35.66	15.99±19.63	
		Week12	6.72±8.19	20.76±36.96 a	27.11±7.49*b	**0.005**
	**Total walnuts, g/d**	Baseline	3.53±4.84	12.72±33.01	8.05±8.85	
		Week12	3.19±5.03	16.5±35.05 a	23.04±7.95*b	**0.001**

	**Vegetables, g/d**	Baseline	241.95±221.96	268.1±121.34	315.21±169.62	
		Week12	257.17±221.8	290.34±133.72	362.13±132.74 *	0.324
	**Legumes, g/d**	Baseline	14±9.93	15.4±10.94	20.33±10.64	
		Week12	14±9.93	17.84±13.97 a	32.14±14.17*b	**0.003**
	**Fruits, g/d**	Baseline	137.50±75.78	366.41±232.63	309.45±257.58	
		Week12	141.07±122.05	418.84±243.70	507.19±359*	0.135
	**Cereals, g/d**	Baseline	75.63±41.49	167.57±97.01	157.72±88.69	
		Week12	76.06±41.86	152.95±101.79 a	116.8±56.75*b	**0.012**
	**Whole cereal, g/d**	Baseline	25.02±33.7	67.43±71.16	72.36±91.69	
		Week12	35.4±34.04	62.87±58.75	72.55±63.96	0.57
	**Refined cereal, g/d**	Baseline	50.61±36.98	100.14±96.82	85.35±61.66	
		Week12	40.66±25.88	90.08±82.64 a	44.25±37.84*	0.055
	**Fish or seafood, g/d**	Baseline	113.32±82.17	83.11±38.08	99.23±54.71	
		Week12	111.35±81.98	99.51±53.46	126.38±51.92*	0.192
	**Blue fish, g/d**	Baseline	9.08±8.31	16.15±14.76	21.29±22.33	
		Week12	9.08±8.31	19.25±24.29 a	41.78±28.46*	**0.025**
	**White fish, g/d**	Baseline	40.96±25.9	24.76±23.71	31.51±29.31	
		Week12	40.96±25.9	28.85±26.45	39.23±28.62*	0.448
	**Meat or meat products, g/d**	Baseline	180.66±28.69	168.5±72.13	135.09±66.01	
		Week12	166.03±50.47	156.76±70.46	110.31±55.46	0.549
	**Red meat, g/d**	Baseline	53.57±30.98	43.13±38.77	31.71±24.43	
		Week12	50.56±33.56	38.38±31.59	20.75±21.85*	0.223
	**White meat, g/d**	Baseline	83.46±44.43	91.25±24.79	77.86±40.76	
		Week12	71.08±47.24	88.88±28.17	70.6±32.59	0.871
	**Processed meat, g/d**	Baseline	43.63±27.77	30.65±26.91	24.49±19.15	
		Week12	44.4±27.19	27.19±26.36	17.93±12.19	0.366
	**Sweets, g/d**	Baseline	47.65±34.27	35.44±39.49	38.15±29.9	
		Week12	44.84±36.68	23.17±34.3*	25.04±25.64*	0.469
	**Dairy products, g/d**	Baseline	260.95±265.25	214.1±253.51	252.21±282.93	
		Week12	266.9±262.06	187.06±159.27	283±277.55	0.774
	**Alcohol, g/d**	Baseline	202.78±233.59	118.31±179.82	149.15±218.19	
		Week12	199.85±233.83	110.17±181.84 a	168.91±210.2	**0.046**
	**Wine, mL/d**	Baseline	121.43±192.67	28.46±33.05	47.37±52.5	
		Week12	119.05±193.92	21.55±22.87	59.75±67.95	0.469
	**MEDAS**	Baseline	5±0.89	7.95±1.50	7.96±2.30	
		Week12	5.33±0.82	8.05±0.81 a	11.96±1.13*b	**<0.005**
**Key food items**						
	**Energy, kcal/d**	Baseline	2805.64±521.7	2425.21±724.16	2465.94±843.83	
		Week12	2536.55±535.24	2326.16±497.11	2507.14±729.81	0.193
	**Protein, g/d**	Baseline	100.34±26.89	99.13±30.41	94.13±30.26	
		Week12	97.06±29.56*	97.38±30.38	92.42±22.61	0.15
	**Carbohydrate, g/d**	Baseline	256.71±94.71	273.93±110.06	291.8±80.95	

	Week12	240.85±97.59	265.6±116.35	250.17±61.32*	0.873
**Fibre, g/d**	Baseline	19.98±5.59	27.4±11.24	23.58±13.05	
	Week12	21.22±8.48	23.71±8.75	23.18±6.15	0.093
**Soluble fibre, g/d**	Baseline	1.62±1.04	1.96±1,37	2.06±1.9	
	Week12	1.55±0.84	1.95±1,18	1.99±1.28	0.873
**Total fat, g/d**	Baseline	122.82±39.68	115.77±36.25	111.68±31.01	
	Week12	131.53±35.28	115.9±35.67	118.3±19.15	0.273
**SFA, g/d**	Baseline	37.47±18.46	29.2±11.48	29.32±12.98	
	Week12	38.78±17.51	27.11±7.77	25.83±8.32	0.396
**MUFA, g/d**	Baseline	53.07±16.89	54.93±13.44	52.06±14.27	
	Week12	58.69±13.65	54.54±12	57.12±7.97*	0.12
**PUFA, g/d**	Baseline	16.22±4.24	17.93±14.51	17.45±4.84	
	Week12	16.93±3.78	20.22±15.79	22.67±3.91*b	0.174
**Linoleic acid, g/d**	Baseline	13.94±6.55	10.12±3.87	12.36±6.84	
	Week12	11.18±5.27	9.95±3.68	10.78±5.61*b	0.352
**α-linolenic acid, g/d**	Baseline	1.74±0.88	1.47±1.01	1.69±1.23	
	Week12	1.28±0.61	1.76±1.17 a	2.01±1.14*b	0.217
**Cholesterol, mg/d**	Baseline	116.91±48.12	81.18±35.56	96.02±72.06	
	Week12	98.03±35.23	70.02±22.41	90.81±64.01	0.508
**Sugar, g/d**	Baseline	483.55±178.78	349.74±169.71	353.21±147.84	
	Week12	444.56±134.11	313.42±144.46	335.24±162.76	0.715
**Polyphenols, mg/d**	Baseline	816.34±300.24	957.66±654.45	871.30±309.27	
	Week12	799.63±262.32	962.37±427.85	823.12±217.94	0.538
**Flavonoids, mg/d**	Baseline	429.40±307.24	620.12±658.77	515.72±247.98	
	Week12	430.80±255.94	595.72±361.02	473.35±156.17	0.23
**Phenolic acids, mg/d**	Baseline	317.09±166.28	254.33±130.27	287.03±126.63	
	Week12	297.27±161.76	288.01±140.22	279.16±126.82	0.209
**Stilbenes, mg/d**	Baseline	2.87±5.29	1.06±1.39	1.32±1.78	
	Week12	3.18±5.51a	1.21±2.17b	1.04±1.25b	0.154
**Lignans, mg/d**	Baseline	1.06±0.44	1.35±0.47	1.97±0.57	
	Week12	1.47±0.56*a	1.46±0.53b	2.21±0.64*c	**0.049**
**Other polyphenols, mg/d**	Baseline	65.92±22.39	80.80±41.22	65.26±29.45	
	Week12	66.91±18.34	75.97±39.72	75.97±39.72	0.879

Supplementary Table 4. Inflammatory, bacterial translocation and immunological markers in High-Adherence (n=9) and Low-Adherence (n=31) groups at baseline. Mann-Whitney-Wilcoxon test with Bonferroni was used to examine parameters between Adherence extreme groups. P<0.05 was considered significant; not significantly significant features between groups were detected. Acronyms: uRCP, ultrasensitive c-reactive protein; IL-6, interleukin-6; DD, D-dimer; sCD14, soluble CD14; LBP, lipopolysaccharide binding-protein; Treg, regulatory T-cell.

**Marker**	**Low-Adherence at baseline**	**High-Adherence at baseline**	**P-value**
**Inflammation**			
PCR	0.27	0.22	0.473

IL6	3.90	3.67	0.438
DD	253.33	422.22	0.107
**Bacterial translocation**			
sCD14	1735.39	1653.89	0.987
LBP	11417.76	13081.04	0.180
**Immune activation**			
CD4+ (%)	48.28	47.99	0.897
CD8+ (%)	43.41	44.40	0.799
CD4+HLADR+CD38+ (%)	2.25	1.65	0.750
CD8+HLADR+CD38+ (%)	3.65	4.12	0.799
**Treg cells**			
CD4+Foxp3+CD25+ (%)	4.65	3.94	0.206
CD4+Foxp3+CD25+bright (%)	2.14	1.86	0.507
CD4+Foxp3+CD25- (%)	3.78	3.71	0.373
CD4+Foxp3+CD25+CD127- (%)	4.06	3.41	0.199
CD4+Foxp3+CD25+CD127+ (%)	0.59	0.48	0.248
CD4+Foxp3+CD25+brightCD127- (%)	1.96	1.68	0.371
CD4+Foxp3+CD25+brightCD127+ (%)	0.18	0.16	0.639
**T-helper 17 (Th17) cells**			
CD4+IL17A+ (%)	0.84	0.71	0.354
CD4+IFNg+ (%)	18.40	25.86	0.093
CD4+IL17A+IFNg+ (%)	0.15	0.17	0.891
CD8+IL17A+ (%)	0.02	0.02	0.830
CD8+IFNg+ (%)	58.60	57.46	0.711
CD8+IL17A+IFNg+ (%)	0.05	0.03	0.355

Supplementary Table 5. Markers in High-Adherence and Low-Adherence groups in MSM individuals. Metabolic, inflammation, bacterial translocation, immunological and nutrition markers in High-Adherence and Low-Adherence MEDAS groups. MSM individuals were included (n = 60). Acronyms: hdl, high-density lipoprotein; ldl, low-density lipoprotein; uRCP, ultrasensitive c-reactive protein; IL-6, interleukin-6; DD, D-dimer; sCD14, soluble CD14; LBP, lipopolysaccharide binding-protein; Treg, regulatory T-cell; BMI, body mass index. Mann-Whitney-Wilcoxon test with Bonferroni was used to examine parameters between Adherence extreme groups. *P value for the difference between baseline and week 12 values (paired). **P value for the difference between delta and ratios values of both groups (unpaired). P<0.05 was considered significant and highlighted in bold type.

**MSM (n = 60)**								
		**Low-Adherence (n = 8)**			**High-Adherence (n = 30)**			**Δ Low vs High**
**Parameters**	**Markers**	Baseline	week12	P *	Baseline	week12	P *	P **
**Metabolic**								
	**Glucose (mg/dL)**	84 (78, 92)	81 (73.5, 96)	0.88	82.5 (77, 91.25)	90 (83, 99.25)	0.09	0.57
	**Creatinine (mg/dL)**	0.89 (0.8, 1.05)	0.82 (0.79, 0.9675)	0.63	0.95 (0.8, 1.06)	0.91 (0.86, 0.95)	0.18	0.81
	**Cholesterol (mg/dL)**	167.5 (159.5, 195.8)	190.5 (154.5, 206.5)	0.54	168 (154, 197)	172.5 (161.2, 192)	0.67	0.69
	**Hdl (mg/dL)**	41 (34.75, 49)	41 (37, 53.25)	0.18	37 (33, 44)	38.5 (34.75, 43.75)	0.54	0.45

**Ldl (mg/dL)**	106 (101.8, 115.5)	115.5 (86.25, 133.25)	1	114 (90, 121)	112 (106, 130)	0.56	0.86
**A1-lipoprotein (mg/dL)**	140 (135, 149)	132 (121.8, 152.5)	0.94	126 (116, 131.5)	132 (121, 137)	0.17	0.66
**B-lipoprotein (mg/dL)**	85 (82, 101)	100 (87.75, 110.5)	0.09	96 (81.5, 112.5)	94 (87, 111)	0.55	0.24
**Triglycerides (mg/dL)**	84.5 (64.5, 131)	89.5 (71.75, 114)	0.44	118 (72.5, 145.2)	97 (75.75, 154)	0.69	0.32
**Alkaline phosphatase (U/L)**	67 (57, 83.25)	69 (64, 81.75)	0.84	76.5 (66.75, 97.25)	73.5 (69.75, 103.25)	0.58	0.82
**Asat (U/L)**	21.5 (19.5, 25.5)	23 (20, 25.5)	0.64	27.5 (23, 29.25)	23.5 (21.75, 26.25)	**0.02**	0.06
**Alat (U/L)**	23.5 (16.25, 30.75)	21 (19, 30.75)	0.55	27 (20, 30.25)	26(18.75, 30.5)	0.09	0.15
**Bilirubin-total (mg/dL)**	0.75 (0.52, 0.975)	0.6 (0.43, 0.85)	0.76	0.6 (0.5, 0.7)	0.55 (0.4, 0.7)	0.42	0.98
**Platelets (10^9/L)**	202 (180.5, 243.8)	227 (215, 235.2)	**0.04**	219.5 (189, 250.5)	234 (180.2, 264)	1	0.11
**Leucocytes (10^9/L)**	7.67 (6.16, 7.94)	6.53 (5.54, 7.105)	**0.05**	7 (5.8, 8.38)	6.06 (5.46, 7.205)	0.1	0.54
**Hematocrit (L/L)**	0.45 (0.43, 0.46)	0.45 (0.44, 0.465)	0.72	0.46 (0.43, 0.49)	0.44 (0.44, 0.4625)	0.48	0.41
**HIV infection**							
**Viral load (cp/mL)**	undetectable	undetectable	-	undetectable	undetectable	-	-
**CD4+ T-cells (cell/mm3)**	827 (601.2, 1082)	892.5 (699.8, 1034.8)	0.72	783 (641.8, 996)	733.5 (636.5, 904.2)	0.11	0.79
**Inflammation**							
**uRCP (mg/dL)**	0.1 (0.06, 0.18)	0.08 (0.04, 0.18)	0.35	0.16 (0.09, 0.2525)	0.19 (0.12, 0.4925)	0.09	0.09
**IL-6 (pg/dL)**	2 (2, 3)	2 (2, 3)	0.27	2 (2, 5)	2 (2, 2.5)	0.96	0.76
**DD (ng/dL)**	200 (150, 300)	200 (150, 300)	1	200 (150, 300)	200 (200, 200)	0.96	0.5
**Bacterial translocation**							
**sCD14 (ng/dL)**	1571 (1440, 1710)	1533.4 (1373.2, 1802.8)	0.65	1770.4 (1574.9, 1888.6)	1743 (1515, 1850)	0.83	0.56
**LBP (ng/dL)**	10602 (9714, 13942)	11706 (9579, 13297)	0.5	10491 (9140, 14609)	11249 (9412, 14904)	0.99	0.62
**Endocab (MMU/mL)**	51.6 (47.5, 127.05)	55.95 (47.8, 113.4)	0.31	50.9 (27.12, 75.35)	38.6 (28.98, 95.62)	0.85	0.23
**Immune activation**							
**CD4+ (%)**	49.31 (42.53, 64.69)	52.54 (41.40, 62.68)	0.73	43.53 (36.63, 50.83)	47.14 (38.52, 54.23)	0.12	0.82
**CD8+ (%)**	38.22 (32.83, 44.64)	35.21 (32.03, 43.99)	0.65	48.17 (38.59, 55.72)	46.02 (38.70, 53.28)	0.08	0.45
**CD4+HLADR+CD38+ (%)**	1.13 (0.96, 1.53)	1.89 (0.93, 2.47)	0.18	1.15 (0.72, 1.77)	0.99 (0.84, 1.43)	0.35	**< 0.005**
**CD8+HLADRCD+38+ (%)**	2. 00 (1.68, 3.65)	2.58 (2.14, 3.40)	0.13	2.82 (1.85, 4.56)	2.55 (2.11, 4.49)	0.55	**0.007**
**Treg cells**							
**CD4+Foxp3+CD25+ (%)**	4.49 (3.82, 6.39)	4.37 (3.61, 5.945)	0.56	4.39 (3.83, 4.765)	4.22 (3.5, 4.755)	0.55	0.21
**CD4+Foxp3+CD25+bright (%)**	2.05 (1.58, 2.7)	1.92 (167, 2.853)	0.49	1.99 (1.6, 2.25)	1.83 (1.5, 2.292)	0.45	0.4
**CD4+Foxp3+CD25- (%)**	3.77 (3.54, 4.237)	4.15 (3.44, 4.975)	0.49	4.01 (3.27, 4.668)	3.76 (3.07, 5.008)	0.58	0.36
**CD4+Foxp3+CD25+CD127- (%)**	3.9 (3.25, 5.484)	3.84 (3.20, 5.348)	0.49	3.8 (3.14, 4.315)	3.55 (3.04, 4.19)	0.62	0.11
**CD4+ Foxp3+CD25+CD127+ (%)**	0.49 (0.44, 0.6412)	0.56 (0.34, 0.72)	1	0.49 (0.37, 0.7217)	0.49 (0.37, 0.5673)	0.25	0.62
**CD4+Foxp3+CD25+brightCD127- (%)**	1.87 (1.38,2.41)	1.81 (1.5, 2.655)	0.56	1.84 (1.42, 2.1337)	1.72 (1.31, 2.1144)	0.42	0.36
**CD4+ Foxp3+CD25+brightCD127+ (%)**	0.13 (0.12, 0.2362)	0.18 (0.1, 0.19)	0.85	0.18 (0.11, 0.22)	0.16 (0.15, 0.2066)	0.16	0.75

**CD4+Foxp3+CD25-CD127+ (%)**	29.8 (26.66, 38.93)	35.76 (33.11, 40.91)	0.28	38.59 (34.3, 46)	34.91 (29.65, 41.16)	0.2	0.07
**T-helper 17 (Th17) cells**							
**CD4+IL17A+ (%)**	0.48 (0.45, 0.78)	0.57 (0.52, 0.8)	0.82	0.73 (0.38, 1.185)	0.8 (0.55, 1.155)	0.14	0.63
**CD4+IFNg+ (%)**	17.64 (11.34, 20.25)	17.71 (13.24, 21.82)	0.57	20.94 (15.97, 26.08)	19.76 (15.75, 25.43)	0.6	0.9
**CD4+IL17A+IFNg+ (%)**	0.09 (0.06, 0.11)	0.07 (0.06, 0.13)	1	0.13 (0.08, 0.23)	0.14 (0.09, 0.29)	0.65	0.85
**CD8+IL17A+ (%)**	0.01 (0, 0.01)	0.02 (0.01, 0.03)	0.11	0.02 (0.01, 0.025)	0.02 (0, 0.03)	0.47	0.1
**CD8+IFNg+ (%)**	53.27 (44.33 ,69.29)	44.17 (42.67, 64.86)	0.25	61.51 (46.26, 79.7)	50.49 (43.68, 75.47)	0.36	0.65
**CD8+IL17A+IFNg+ (%)**	0.02 (0.02, 0.03)	0.03 (0.01, 0.03)	0.57	0.02 (0.01, 0.05)	0.02 (0.01, 0.04)	0.36	0.41
**Nutrition**							
**BMI**	25 (23, 26.75)	24.5 (23.75, 26)	0.37	25 (23, 27)	25 (24, 27)	1	0.35
**Weight (Kg)**	70.95 (68.05, 77.12)	69.9 (67.65, 78.58)	0.38	74.75 (71.17, 82.38)	74.35 (71.65, 82.6)	0.94	0.29
**Adherence (MEDAS)**	4.5 (4, 5)	5 (4, 6)	1	8 (6, 9.25)	12 (11, 13)	**< 0.005**	**< 0.005**
**Oleic (g/dL)**	17.7 (13.63, 22.06)	17.04 (14.52, 21.94)	1	10.59 (17.84, 23.95)	19.28 (14.34, 23.68)	0.54	0.67
**Linoleic (g/dL)**	21.52 (20.68, 24.14)	21.39 (20.01, 22.65)	0.06	24 (20.48, 28.73)	24.11 (20.3, 30.78)	0.48	0.19
**Alfa-linolenic (g/dL)**	0.22 (0.15, 0.43)	0.22 (0.15, 0.4175)	0.88	0.37 (0.25, 0.492)	0.48 (0.27, 1.3525)	0.01	0.09

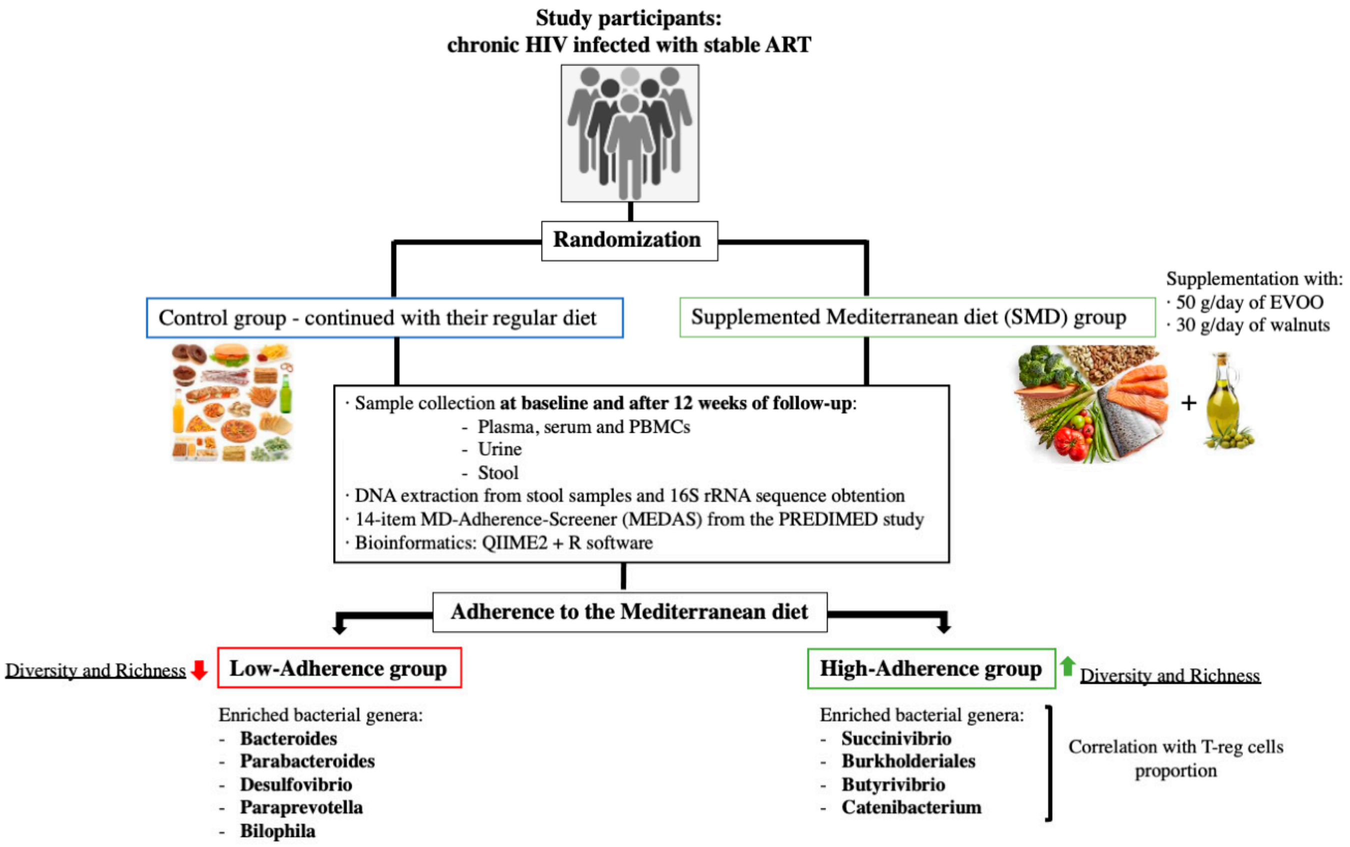

Graphical abstract

Supplementary Figures

Supplementary Figure 1. Schematic description of participants follow-up.

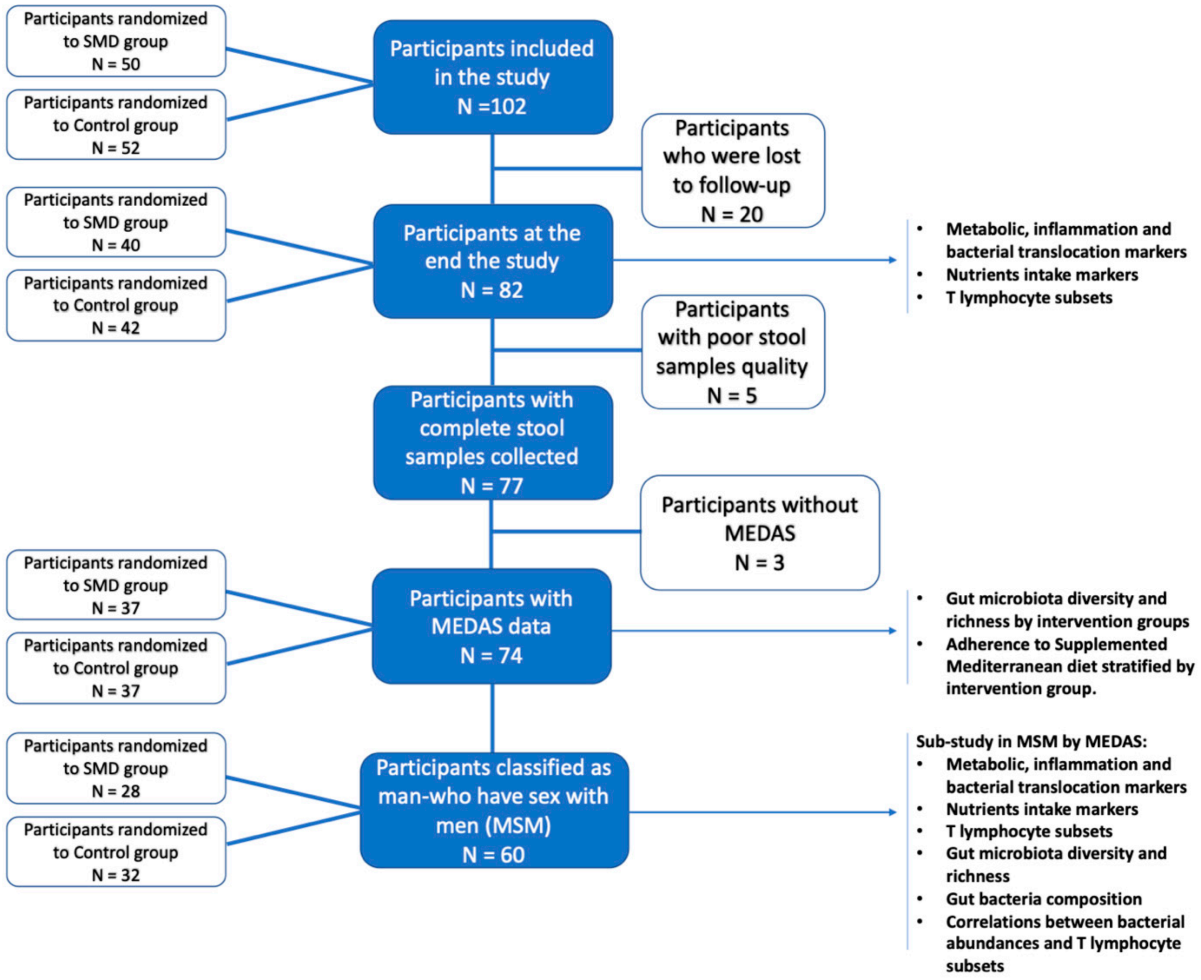

Supplementary Figure 2. Principal Coordinates Analysis (PCoA). Non-metric multidimensional scaling (NMDS) used: Bray Curtis distances. R package: vegan. **a)** Complete cohort adherence at basal time-point. The Low-adherence (**LA**) group is represented by the black squares, n=31; the High-Adherence (**HA**) group is represented by the red squares, n=9. The Adonis (PERMANOVA) test was performed, considering the adherence extreme groups (High and Low adherence): P=0.145, R^2=0.045. **b)** Complete cohort by MD adherence at baseline and at the end of the study. The Low-adherence group at baseline (**LA_basal**) is represented by the black squares, n=31; the Low-adherence at the end of the study (**LA_end**) group is represented by the blue squares, n=11; the High-Adherence group (**HA_basal**) is represented by the red squares, n=9; The High-adherence at the end of the study (**HA_end**) group is represented by the orange squares, n=37. The Adonis (PERMANOVA) test was performed, considering the MD adherence groups (High and Low adherence): P=0.061, R^2=0.060. **c)** MSM individuals by the adherence behaviour to

MD at baseline time-point. The Low-adherence (**LA**) group is represented by the black squares, n=19; the High-Adherence (**HA**) group is represented by the red squares, n=9. The Adonis (PERMANOVA) test was performed, considering the adherence extreme groups (High and Low adherence): P=0.108, R^2=0.076. **d)** MSM individuals by the adherence behaviour to MD at baseline and at the end of the study. The Low-adherence group at baseline (**LA_basal)** is represented by the black squares, n=19; the Low-adherence at the end of the study (**LA_end**) group is represented by the blue squares, n=8; the High-Adherence group (**HA_basal**) is represented by the red squares, n=9; The High-adherence at the end of the study (**HA_end**) group is represented by the orange squares, n=30. The Adonis (PERMANOVA) test was performed, considering the adherence extreme groups (High and Low adherence): P=0.197, R^2=0.074.

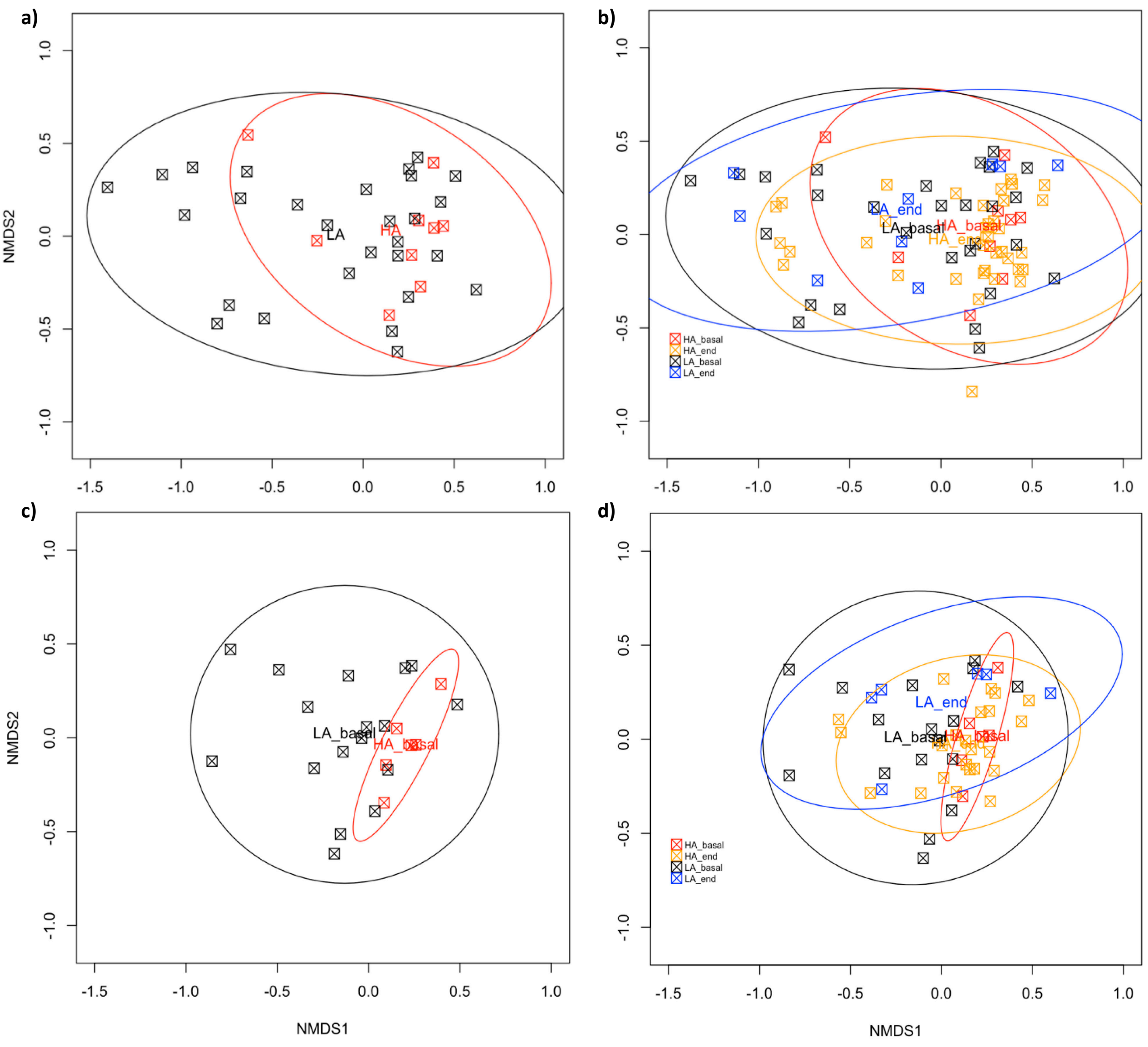

Supplementary Figure 3. Relative abundances (%) according to the randomization into the intervention groups. Relative abundances (%) according to the randomization into the intervention groups. It is presented the microbiota composition at basal and in the end of the study time points. MSM subjects were selected (n =60). Each genus >3% of total relative abundance correspond to a colour linked in the legend.

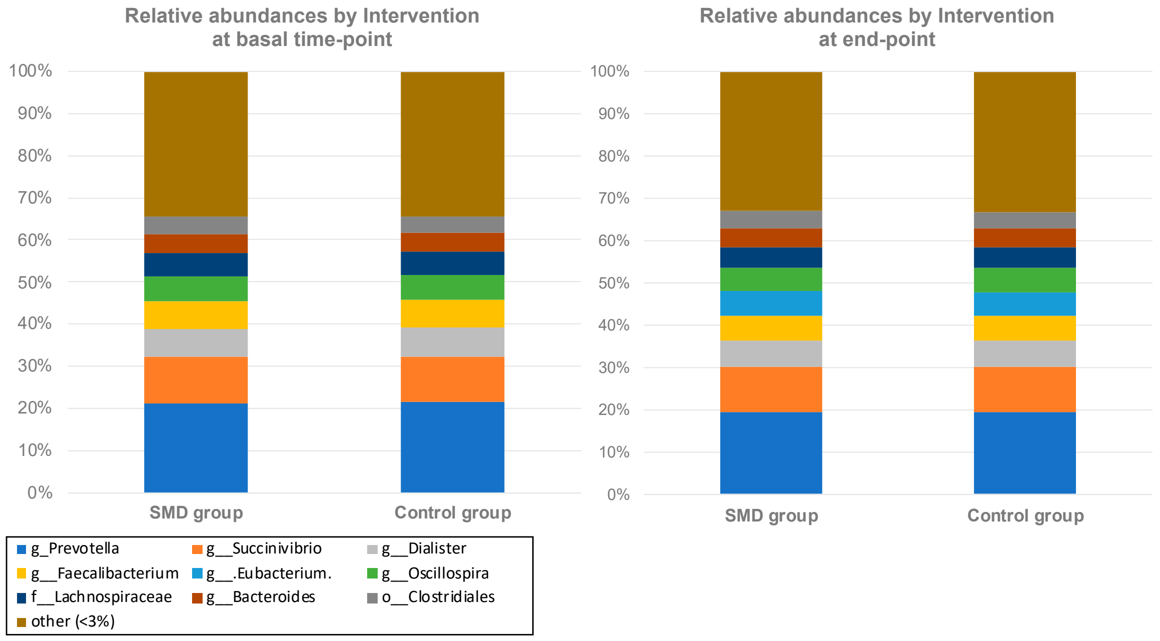

Supplementary Figure 4. Microbiota composition according to the MEDAS in MSM individuals. A) Gut microbiota relative abundances (%) by genus. Subjects were classified according to MEDAS, fixed at week12. It is presented the gut microbiota composition. Number of subjects in: High-Adherence = 30 (5 from Control group and 25 from SMD group), Medium-Adherence = 20 (18 from Control group and 2 from SMD group), Low-Adherence = 8 (all from Control group). Only genus with a relative abundance >3% of total relative abundance are colour-coded. * Mann-Whitney-Wilcoxon test in Bacteroides genus: P=0.0001.

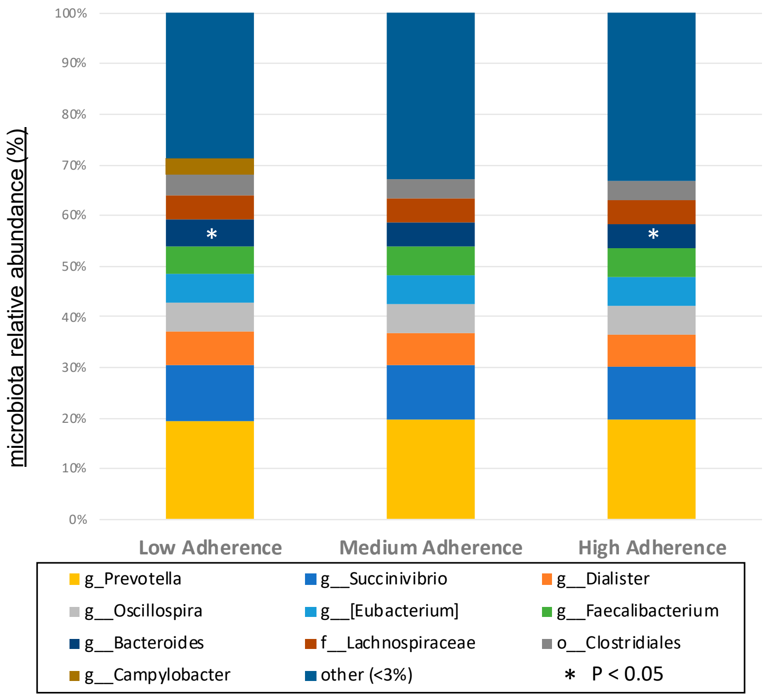

Supplementary Figure 5. Spearman Rho correlation coefficients in ∆Adherence ≧ 4-points group at the end of the study. Each column of the table represents a particular bacterial genus and the rest of parameters analysed in the study (in rows) at week 12 in the group of MSM individuals who presented a ∆Adherence≧4-points (n = 17). Bacterial genera (columns) were grouped by phylum (Actinobacteria, Bacteroidetes, Firmicutes). Only the most significant correlations are shown. Direct (positive) correlations are highlighted in red and inverse (negative) correlations are highlighted in blue. Test applied: Spearman rank correlation with Holm's correction. The corrected P-values were not showed. Levels of statistical significancy: * P ≤0.05, ** P<0.01, *** P<0.005. NA: no significant correlations, P>0.05. The following parameters were analysed: metabolic (Cholesterol, Hdl, Ldl, A1-lipoprotein, B-lipoprotein), inflammation

(uRCP, IL-6, DD), bacterial translocation (sCD14, LBP), nutrition (BMI, Weight, Adherence points (MEDAS), Oleic acid, Linoleic acid, Alfa-linoleic acid, Omega3, Omega6), immune activation (CD4+ T-cells, CD3+CD4+, CD3+CD8+, CD4+HLADR, CD8+HLADR) Treg cells (CD4+Foxp3+CD25, Foxp3+CD25+CD127), IL17 and IFNg production (CD4+IL17A+, CD4+IFNg+, CD4+IL17A+IFNg+, CD8+IL17A+, CD8+IFNg+, CD8+IL17A+IFNg+). Acronyms: Hdl, high-density lipoprotein; Ldl, low-density lipoprotein; uRCP, ultrasensitive c-reactive protein; IL-6, interleukin-6; DD, D-dimer; sCD14, soluble CD14; LBP, lipopolysaccharide binding-protein; Treg, regulatory T-cell; BMI, body mass index.

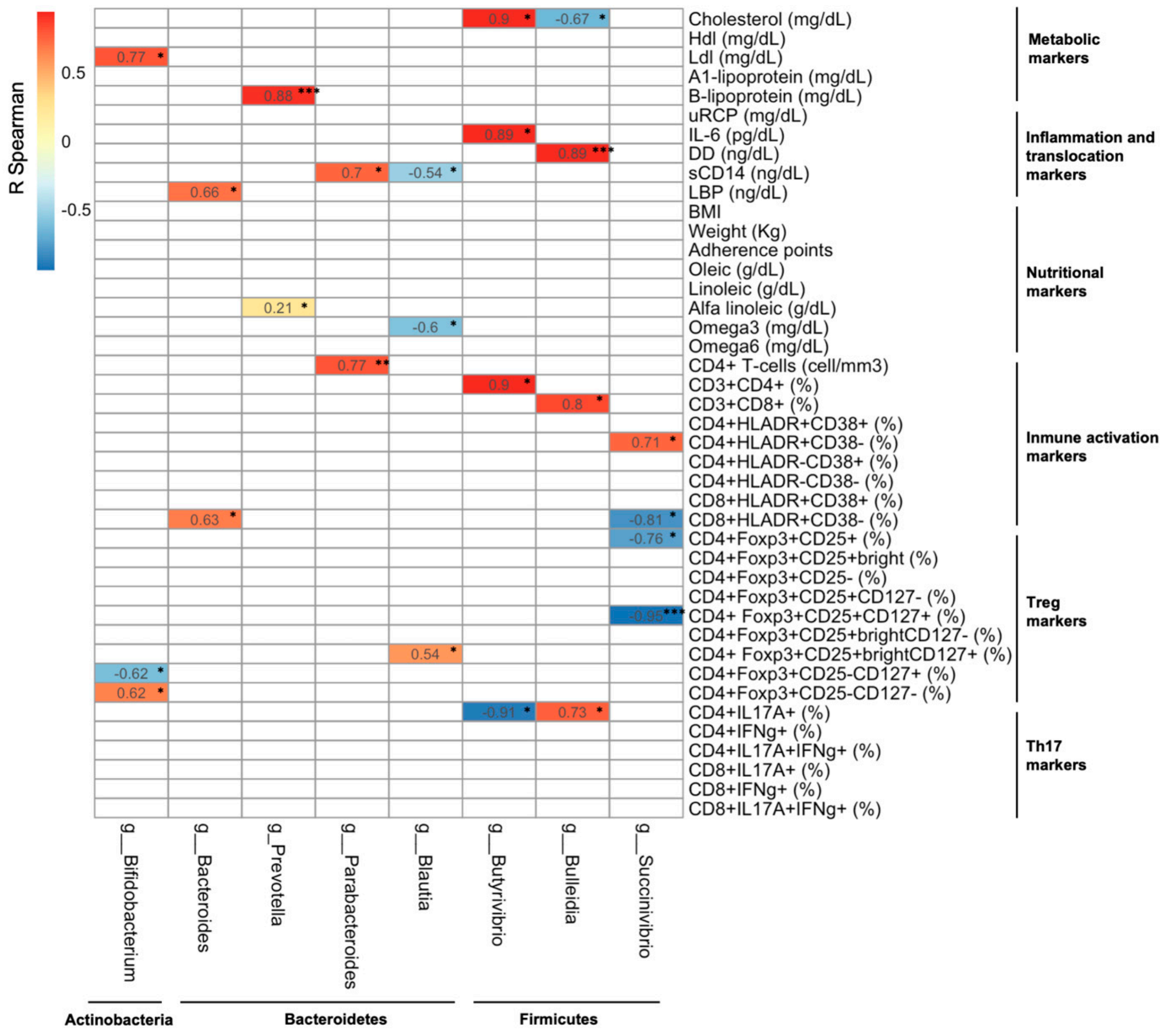

Supplementary Methods

Study Design.

The inclusion criteria were individuals ≥18 years old; on stable ART during the last year before entering the study; nadir CD4 + T-cells ≥ 350 cells/mm^3; CD4 + T-cells at recruitment ≥ 450 cells/mm^3; and undetectable viral load at least during the last 6 months before entering the study. The exclusion criterion was individuals with antibiotics intake in the three months before the beginning of the study.

Participants were advised to eat ab libitum (without energy restriction) and physical activity was not promoted in any of the two groups. In the SMD, participants were encouraged to increase the intake of vegetables, fresh fruit, legumes, nuts, fish or seafood, and to use EVOO for cooking and dressings. A dietitian measured the blood pressure (BP) of the participants in each arm with a validated semiautomatic oscillometer (Omron HEM-705CP, Hoofddorp, the Netherlands), as well as, anthropometric measurements (weight, waist, hip, and height). After, body mass index (BMI) was calculated. In addition, fasting blood, urine, and faeces were collected and stored at -80 ºC until assay.

T lymphocyte subsets analysis.

Activation status and Th17 and Treg cell subsets were analysed by multiparametric flow cytometry on a Cytomics FC500 flow cytometer (Beckman Coulter). For this purpose, three different panels of monoclonal antibodies were designed to stain peripheral blood mononuclear cells (PBMCs). Each panel included 5 different monoclonal antibodies to measure: a) the level of T-cells activation; b) the level of IL17 and IFNγ production by T-cells; c) the level of regulatory T (Treg) cells. To measure the level of activation on T lymphocytes, one million PBMC were surface stained with anti-CD3-PE-Vio615 (Miltenyi, Germany), anti-CD8-PECy5 (Biolegend, USA), anti-CD4-FITC (Biolegend,

USA), anti-HLADR-PE (BD Biosciences, USA) and anti-CD38-PECy7 (Biolegend, USA) by incubating at 4 °C for 30 min. To analyse IL17 (Th17 and Tc17 cell subsets) and IFNγ production, the cytokines IL17 and IFNγ were measured in CD4+ and CD8+ T cells in response to polyclonal stimulation with PMA/ionomicin. Production of IFNγ was used as control of a correct stimulation in the assay. Briefly, one million of PBMCs were cultured in complete medium (RPMI, 10% fetal bovine serum, L-glutamine and antibiotics) and stimulated with PMA/ionomicin (final concentration 50 ng/ml and 1 μM, respectively) during 6 h at 37 °C with 5% CO2. BD GolgiStop (BD Biosciences, USA) and BD GolgiPlug (BD Biosciences, USA) were added during the last five hours of culture. Control condition without stimulation (only medium) was performed for each sample. Then, cells were washed and incubated with anti-CD3-PE-Vio615 (Miltenyi, Germany), anti-CD4-FITC (Biolegend, USA) and anti-CD8-PECy5 (Biolegend, USA) for 30 min at 4 °C. Thereafter, cells were permeabilized using the Cytofix/Cytoperm kit (BD Biosciences, USA) and incubated with anti-IFNγ-PECy7 (BD Biosciences, USA) and anti-IL17A-PE (Biolegend, USA). Th17 cells were defined as CD4+ T cells producing IL17A and Tc17 cells as CD8+ T cells producing IL17A. To analyze Treg cell subsets, levels of FoxP3, CD25 and CD127 were measured in CD4+ T cells. Briefly, one million PBMC were surface stained with anti-CD8-PECy5 (Biolegend, USA), anti-CD4-ECD (Biolegend, USA), anti-CD25-PECy7 and anti-CD127-PE-Vio615 (Miltenyi, Germany) by incubating at 4 °C for 30 min. Then, cells were permeabilized using the 1X FoxP3 Fix/Perm kit (Biolegend, USA) and incubated with anti-FoxP3-PE (Biolegend, USA). Different subsets of CD4+Foxp3+ T cells were analyzed based on the expression of CD25 and CD127 markers. Data analysis was performed using CXP software (Beckman Coulter).

Microbiota composition.

Faecal DNA extraction: faecal samples from study participants were collected using the SOP 03 V2 protocol from the International Human Microbiome Standards (IHMS). Samples were aliquoted and dry cryopreserved at - 80 ºC until DNA extraction. Study participants collected faecal samples in sterile faecal collection tubes on day 0 of the study (baseline) and at week 12 (end of follow-up), following instructions pre-specified on standard operating procedures. Samples were collected and stored at - 80C until assay. The Handbook included in the QIAamp DNA stool mini kit was used. The Inhibitex Tablet (provided by the assay kit) was replaced by two additional steps with ammonium acetate 10M (Sigma-Aldrich, USA) and 1:1 in isopropanol (Sigma-Aldrich, USA). The extracted nucleic acids were quantified using Qubit (Thermo Fisher Scientific, USA), the purity was analysed using Nanodrop 2000 (Thermo Fisher Scientific, USA) and the integrity was examined by agarose 1 % electrophoresis and in the TapeStation system (Agilent 4200; Santa Clara, United States). They were stored at - 20 ºC until the next steps.

Amplicon generation and sequencing: for the amplification of the conserved region V3-V4 from the 16 rRNA gene the next degenerate primers were used: Forward: 5'-ATT GAC GGG GRC CCG CAC-3; Reverse: 5'-CGA GCT GAC ARC CAT GCA-3'. Amplifications were performed in 25 µL reactions, each containing 50 ng of extracted DNA. The amplicons quality control was performed using the 5400 Fragment Analyzer System (Agilent, USA). Amplified DNA templates were cleaned-up for non-DNA molecules and Ilumina sequencing adapters. The sequencing method was performed on an Illumina MiSeqTM platform (Illumina Inc., USA) according to the manufacturer's specifications to generate paired-end reads of ~300 base- length in each direction

Bioinformatic and statistical analysis

Sequence quality control: The quality control of MiSeq raw sequences, the denoising and the trimming process was assessed for the FastQ files using the deblur package of QIIME2 software [1]. Sequences were trimmed using a cutoff of Q = 30 for paired ends. A minimum read length of 190 bp was established.

16S rRNA Sequence Analysis: The Illumina MiSeq raw sequences were converted to multiplexed FastQ format using the q2-demux plugin followed by CASAVA 1.8.2 [2]. Paired-end reads were joined using the QIIME2 2019.10 [1]. Bioinformatic analysis of generated bacterial 16S rRNA data was conducted using the QIIME2 software pipeline. Filtered sequences were aligned and clustered into operational taxonomic unit (OTU) based on the de novo OTU picking algorithm using the q2-mafft - via q2-alignment plugin [3]. Next, chimeras and singletons were removed using q2-uchime plugin followed by *denovo* [4]. The resultant OTU assignation were aligned to generate the phylogenetic trees (rooted and un-rooted) with align-to-tree-mafft-fasttree plugin from QIIME2 software [5]. Taxonomy was assigned to OTUs using the q2-feature-classifier, classify-sklearn plugin, taxonomy classifier against the Greengenes 13_8 99% OTUs reference sequences and Silva 132 99% OTUs [1]. Alpha-diversity metrics (observed OTUs and Faith's Phylogenetic Diversity [6]), beta diversity metrics (weighted UniFrac, unweighted UniFrac distances) [7], Jaccard distance, and the most used non-metric multidimensional scaling (NMDS) Bray-Curtis dissimilarity [8], and Principle Coordinate Analysis (PCoA) were estimated using q2-diversity after samples were rarefied (subsampled without replacement) to 1000 sequences per sample. Downstream data analysis was performed with a combination of QIIME2 and R softwares, using the following R packages: vegan, phylloseq, dplyr, magrittr, ggpubr and dunn.test, for statistical analysis; ggplot2 and corrplot, for data ploting. The following test were used

when required: Wilcoxon's test, Kruskal-Wallis test, Dunn's test, PERMANOVA (QIIME2; diversity beta-group-significance plugin), Adonis, and selbal [9], Linear Discriminant Analysis (LDA) Effect Size (LEfSe), multivariant linear regression, Spearman rank correlation with Holm's correction for multiple comparisons.

Alpha and beta diversity: OTUs with 99 % similarity level were selected for taxonomical assignment using the Greengenes database. The assigned taxonomy was used in the alpha diversity and richness analysis (Observed OTUs, Faith's PD, Shannon, Simpson, Fisher index, Evenness (Pielou). Alpha diversity calculation was performed also with QIIME2 for Faith's PD and total observed OTUs and R software for the rest of metrics. Beta diversity were calculated within QIIME2 and R software using Bray Curtis, Unifrac and Euclidean distances. From this metrics, the principal coordinates analysis (PCoA) were performed and PCoA plots were obtained into two and three-dimensions. To evaluate the similarities between bacterial communities only MSM were selected. Samples were stratified into subgroups according to: intervention groups (SMD or Control), time-point (basal, week 12) and the MEDAS groups (High-Adherence (MEDAS $\geq$ 10), medium-Adherence (MEDAS 7-10), Low-Adherence (MEDAS < 7); numeric parameters were divided into groups according to the median and quartiles (Q1 and Q3), for each variable along the samples.

Genus abundance: To assess genus abundance, OTU counts were collapsed to the bacterial phylum and genus level. Genus proportion were calculated for each sample. For the differential genus abundance, the Kruskal-Wallis and Dunn's post-hoc test were used to compare groups. Only values became statistical significance after Bonferroni correction [10], considering $P < 0.05$. Taxa abundance composition bar plots were obtained using QIIME2 software. Phylum or genus composition of each individual were represented in consecutive bar plots ordered by the different subgroups cited above.

Balances between subgroups were obtained using gneiss plugin from QIIME2, next steps were executed in R software.

Supplementary references

1. Bolyen E, Rideout JR, Dillon MR, et al. Reproducible, interactive, scalable and extensible microbiome data science using QIIME 2. Nat Biotechnol. **2019**; 37(8):852–857.
2. Allali I, Arnold JW, Roach J, et al. A comparison of sequencing platforms and bioinformatics pipelines for compositional analysis of the gut microbiome. BMC Microbiol. BMC Microbiology; **2017**; 17(1):1–16.
3. Katoh K, Rozewicki J, Yamada KD. MAFFT online service: Multiple sequence alignment, interactive sequence choice and visualization. Brief Bioinform. **2018**; 20(4):1160–1166.
4. Edgar RC, Haas BJ, Clemente JC, Quince C, Knight R. UCHIME improves sensitivity and speed of chimera detection. Bioinformatics. **2011**; 27(16):2194–2200.
5. Price MN, Dehal PS, Arkin AP. FastTree 2 - Approximately maximum-likelihood trees for large alignments. PLoS One. **2010**; 5(3).
6. D.P. F. SYSTEMATICS AND CONSERVATION: ON PREDICTING THE FEATURE DIVERSITY OF SUBSETS OF TAXA. Cladistics. **1992**; .
7. Lozupone C, Lladser ME, Knights D, Stombaugh J, Knight R. UniFrac: An effective distance metric for microbial community comparison. ISME J. Nature Publishing Group; **2011**; 5(2):169–172.
8. Schwendner P, Mahnert A, Koskinen K, et al. Preparing for the crewed Mars journey: microbiota dynamics in the confined Mars500 habitat during simulated

Mars flight and landing. Microbiome. Microbiome; **2017**; 5(1):129.

9. Rivera-Pinto J, Egozcue JJ, Pawlowsky-Glahn V, Paredes R, Noguera-Julian M, Calle ML. Balances: a New Perspective for Microbiome Analysis. mSystems. **2018**; 3(4):1–12.

10. Lee S, Lee DK. What is the proper way to apply the multiple comparison test? Korean J Anesthesiol. **2018**; 71(5):353–360.
